# Lectures on the Eruptive Fevers, Delivered at St. Thomas's Hospital, in January 1843

**Published:** 1844-01-01

**Authors:** 


### LECTURES ON THE ERUPTIVE FEVERS, DELIVERED AT ST. THOMAS'S HOSPITAL IN JAN. 1843. By *George Gregory*, M.D., &c. London, 1843.

THE great experience which twenty years of official connexion with the Small-pox and Vaccination Hospital were supposed to have given to Dr. Gregory, pointed him out as a person peculiarly well qualified to deliver a course of lectures on the Exanthemata, a class of diseases, whose pathology, and more especially the diagnosis, oftentimes proves so embarrassing to the student, as well as to the junior practitioner. That the selection was a judicious one, will, we think, appear evident from the succinct analysis we now propose to give of the volume before us. The lectures, in their published form, are thirteen in number. In the *First* of these are considered the *Character and Affinities of the Eruptive Fevers*. The author commences by considering the relation in which the exanthemata stand; 1, to each other; 2, to other forms of fever; 3, to other diseases of the superficies; 4, to other diseases arising from morbid poison; 5, to the diseases of other structures. The greater exanthemata, by which he means those that bring life into danger, are four in number: *small-pox, measles, scarlatina, fever*, and *erysipelas*. On the subject of the exanthematic or epidemic mortality—its amount—the proportion which deaths by the exanthemata bear to the deaths by all other diseases—the constancy or inconstancy of this proportion, and other similar points, he says:—

"Upon an average of years, 350,000 persons die annually throughout England and Wales, and 46,000 in the metropolis. The mortality by the four great epidemic maladies (small-pox, measles, scarlatina, and hooping-cough), is very nearly 40,000 in England and Wales, and about 5000 in the metropolis, averaging one in nine of the total mortality, or eleven per cent. This is a very large proportion. That four diseases only should absorb one-ninth of the total mortality of this, and probably of all other countries, may well excite our surprise."

If, says the author, the exanthemata are considered independently of the hooping-cough, considerable fluctuations will be perceived, the mortality by them sometimes falling as low as six per cent, at times rising to near thirteen; but a very important principle comes into play here, which serves to equalise the amount of epidemic mortality. This curious doctrine had long been surmised, but was never proved until the statistical enquiries of recent times shewed its correctness. We may, he says, for want of a better name, call it the law of vicarious mortality, by which is understood, that whenever one epidemic diminishes, another increases, so that the sum-total of epidemic mortality remains, on an average of years, nearly the same. The following table exemplifies this:—

*Table, exhibiting the Amount of Epidemic Mortality in England and Wales, during the years* 1838, 1839, 1840.

| | Year 1838. | Year 1839. | Year 1840. |
|---|---|---|---|
| Small-pox .. .. .. .. | 16,268 | 9,131 | 10,434 |
| Measles .. .. .. .. .. | 6,514 | 10,937 | 9,326 |
| Scarlet Fever .. .. .. .. | 5,802 | 10,325 | 19,816 |
| Total Mortality by the Exanthemata .. | 28,584 | 30,393 | 39,576 |
| Hooping-cough .. .. .. | 9,107 | 8,165 | 6,132 |
| Total of Epidemic Mortality .. .. | 37,691 | 38,558 | 45,708 |
| Total Mortality throughout England and Wales .. .. .. .. | 342,529 | 338,979 | 359,561 |

This table shews that every year is distinguished by some master epidemic. In 1838, small-pox was the ruling epidemic throughout England. In 1839, measles and scarlatina struggled for the mastery. In 1840, scarlet fever was so general and so fatal that the mortality by it exceeded by one-fifth the ravages of small-pox during an epidemic season, (1838,) and more than doubled the mortality by that disease in 1839.

The following table, exhibiting the amount of epidemic mortality in the metropolis during a period of five years, shews that the same general principle applies to town and country, but is less manifest in the smaller population:—

*Table, shewing the Amount of Epidemic Mortality in London, during Five Years*—1838 *to* 1842.

| | Year 1838. | Year 1839. | Year 1840. | Year 1841. | Year 1842. |
|---|---|---|---|---|---|
| Small-pox .. .. .. .. .. | 3,817 | 634 | 1,235 | 1,053 | 360 |
| Measles .. .. .. .. . | 588 | 2,036 | 1,132 | 973 | 1,292 |
| Scarlet Fever .. .. .. .. | 1,524 | 2,499 | 1,954 | 663 | 1,224 |
| Total Mortality by the Exanthemata .. .. .. | 5,929 | 5,169 | 4,321 | 2,689 | 2,876 |
| Hooping-cough .. .. | 2,083 | 1,161 | 1,069 | 2,278 | 1,603 |
| Total of Epidemic Mortality | 8,012 | 6,330 | 5,390 | 4,967 | 4,479 |
| Total Mortality throughout London .. .. .. .. | 52,698 | 45,441 | 46,281 | 45,284 | 45,272 |

From this Table it appears that, in 1838, Small-pox was the great epidemic in London, as in the country. In 1839, measles and scarlet fever were both on the increase, while small-pox had sunk from 3,817 to 634. In 1840, scarlet fever predominated. In 1841, hooping-cough doubled its numbers, and shot above all the rest; while scarlet fever sunk to the low point which small-pox had reached in 1839.

You will perceive from all this, says the author, that vaccination, great as its merits are, does not, and cannot do all that its too sanguine admirers wish. The blessings of vaccination are met and counterbalanced by the law of vicarious mortality. How, and why is this? The explanation is easy. The weak plants of a nursery must be weeded out. If weakly children do not fall victims to small-pox, they live to fall into the jaws of tyrants scarcely less inexorable. Surely Dr. Gregory is not serious in saying that the blessings of vaccination are counterbalanced by the law of vicarious mortality. His attempted explanation of why the thing is so, we do not at all like. Certain it is that the sum of human misery has been considerably diminished by the introduction of vaccination. Dr. Haygarth, who entertained some extravagant ideas of effecting the annihilation of small-pox by universal inoculation, calculated, that if the thing could be accomplished, in fifty years more than one-eighth would be added to the population.

The general character of the exanthemata is derived from the following sources: 1. From the presence and course of the accompanying fever. 2. From the course of the eruption. 3. From the law of universal susceptibility. 4. From the law of non-recurrence. 5. From the law of contagious origin. 6. From the law of epidemic diffusion. With respect to the character of the accompanying fever, in the case of the exanthemata, it is almost uniformly inflammatory. It is found to be so in nineteen-twentieths of the cases of small-pox and measles. The low or nervous form of fever occasionally characterises scarlatina and erysipelas. The malignant form of fever is occasionally witnessed in small-pox and scarlatina.

The notion that "fever precedes the specific action of the exanthematous poisons," has prevailed at all times, and still holds its ground; it is adhered to by Dr. Williams, in his work on Morbid Poisons. He calls fever the *primary* effect of the poison; affection of the skin and mucous membranes, he calls the *secondary* effect of the poison; and the inflammation of internal organs its *tertiary* effect. From this doctrine our author entirely dissents, his opinion being, that exanthema may take place without fever, that the febrile state is not essential to the development of the exanthem. "For," he says, "cow-pox, varicella, inoculated small-pox, and the mildest type of scarlatina, frequently display themselves without initiatory, without eruptive, nay, even without maturative fever." In fact, he says, "the less the fever, the more perfectly is the eruption developed, and the more normal is the course of the disease. Any tumultuous febrile action disturbs the regular progress of an exanthem. Witness scarlatina with excessive angina. There is here literally no eruption at all. We call the complaint angina maligna. Witness the recession of the eruption in malignant measles. Witness the ill-developed eruption of petechial small-pox."

Dr. G. Gregory appears to us to mistake the real question in this place, and, from so doing, to expend a great deal of words to no purpose whatever. We think the question to be this, whether the existence of fever is essential to the existence of a certain disease caused by a specific poison, which disease is, in most cases, but not invariably, characterised by an eruption, as one of its leading symptoms. The question which Dr. Gregory is discussing is, whether the existence of fever is essential to the existence of exanthem. We think that in all cases of the greater exanthemata there is always more or less of fever; but we do not think that the completeness of the eruption depends on the violence or intensity of the fever; nay, we see that where the eruption is most complete, the violence of the fever is least. What we mean is, that there is not that connexion between the fever and the eruption, as that they should bear a direct ratio, the one to the other. Our opinion is that a certain coincidence is to be observed between a certain amount or form of eruption and a certain character of fever. We do not think that either is caused by the other, but that they each, severally, depend on the nature of the exanthematous poison, as well as, probably, on the constitution of the individual. In this way, probably, Burserius, Vogel, De Haen, Frank, and those other credulous mortals, whom Dr. G. Gregory takes to task for avowing their belief in such a monstrous phenomenon as "the Irish mode of undergoing small-pox" without an eruption, would endeavour to excuse their credulity. They, like ourselves, would very likely make so bold as to tell him that they considered the eruption only as a symptom of the disease, not the disease itself.

In speaking of the second character of the exanthemata, viz. the eruption, he notices a doctrine recently brought forward under the title of the *symmetry of diseased action;* by which it is understood that in disease both sides of the body are affected alike. The chief illustrations of the symmetry of disease are to be found in the phenomena of rheumatism, in the mode in which the teeth decay, in the growth of certain tumors; but best of all, in the appearance of the exanthematic eruption. In the corymbose form of small-pox the patches, or corymbi, will be found to correspond on the two sides of the body in the most extraordinary manner.

With respect to the primary sources of the exanthematic poisons, it was believed by several eminent men, long before the discovery of vaccination, that they were originally derived from cattle.

In Lecture Second, the author considers *the Character and Management of the Eruptive Fevers.*

The third character of the Eruptive Fevers our author derives from the law of universal susceptibility; there being no principle more generally recognised than that small-pox and measles necessarily and unavoidably occur to every man once in the course of life. There are, however, some countries in the world not yet visited by the exanthemata. Small-pox, measles, and scarlet fever are to this day unknown in Australia and Van Dieman's Land. The fourth peculiarity of the eruptive fevers is derived from the law of non-recurrence. Immunity from second attacks of the same disease is a very important principle in pathology, applying not merely to small-pox, measles and hooping cough, but to all diseases whatever, which originate from a poison or miasm. It belongs, though in a less degree, to fevers of *endemic* origin—as ague and remitting fever. It is even traceable in fevers of internal origin, as gout and rheumatism. In all cases, therefore, the susceptibility of a disease is more or less exhausted by once undergoing it. A gradation in this respect may easily be traced from rheumatism and gout, (where the law obtains least.) through ague and every variety of endemic fever, whether remittent or continued, up to plague, scarlet fever, yellow fever, measles and small-pox. The law of non-recurrence is more strikingly displayed in measles and small-pox than in any other known disorder. Exceptions occur, however, even here.

We now come to the fifth characteristic of the exanthemata—contagious origin. The contagious, infective miasm, or *materies morbi,* obtains access to the human body in three modes. First, by the inhalation of air tainted by the breath or perspiration of a patient. This is called the mode of infection. Small-pox, measles, plague, typhus, scarlatina, and erysipelas, are thus communicated. Secondly, miasms gain access to the body by solution in the fluids, or humors, and subsequent application to the unbroken surface. It is in this way that psora, tinea capitis, gonorrhœa and the venereal disease are communicated from man to man. This mode is called contagion, *a contactu corporis.* The materies morbi, however, must be dissolved. The germ of disease is conveyed in the fluid form into the interior of the frame, where it mixes with and taints the blood. Thirdly, some of the morbid poisons are not admitted into the frame unless they are applied to an abraded or wounded surface. Hydrophobia, vaccinia, and farcinoma, (or glanders) are received in this way. Small-pox, and plague may thus be excited artificially, and the process is called inoculation. Direct communication of disease by means of the blood, and not by the secretion from the blood, is one of those points in pathology which is now attracting considerable attention on the Continent. The injection of the blood of a glandered horse into the veins of a healthy horse communicates the disease. Measles has been communicated by inoculation with the blood in very many instances. Each specific miasm has its respective laws—its period of latency, of development, and of decline. With reference to the period of incubation, the morbid miasms are divisible into three classes:—1st. Those of *rapid* incubation—viz. chicken-pox, plague, scarlatina, and gonorrhœa. In these instances, the latent period is less than a week. 2nd. Those of *mature* incubation, the period extending from ten to fourteen days—in this class come small-pox, measles, and hooping-cough. 3rd. Those of *tedious* incubation (extending from four to six weeks), In this class we place hydrophobia and secondary syphilis.

"From the earliest period at which the existence of morbid poisons became known, the analogy of vegetable fermentation has been adduced to explain their *modus operandi.* The doctrine of a fermentative process going on during the incubation of small-pox and measles, was distinctly announced by Sydenham, Willis, Diemerbrock, and Morton. Liebig has lately given increased interest to this portion of pathology, by reviving the hypothesis of fermentation, and investing it with a scientific character."

"The phenomena attending the transformation of organic vegetable compounds afford, he says, not merely an analogy, but a correct explanation of the changes taking place in the animal economy by the agency of morbid poisons." Nothing, however, can be clearer than that in this notion, whether correct or not, Liebig is anticipated by Diemerbroek, who flourished two hundred years ago. "Out of an infected body (says he) flow forth continual streams, which, being received by other bodies, presently ferment with the blood, and excite the latent and homogeneous seeds of the same distemper, disposing them into the idea or character of the same disease."

Mr. Farr, in his fourth report, proposes to call all those diseases, which have the property of communicating their own action, and effecting analogous transformations, zymotic diseases (from ζυμοω, to ferment,) and the action itself, zymosis. Zymotic diseases will comprehend all those now associated under the names of "epidemic, endemic, and contagious maladies." One very remarkable character of the zymotic miasms is, that they operate upon the healthy body without the aid of predisposing causes. A man in the most perfect health contracts small-pox or measles, and this state of body is the best possible for insuring the success of inoculation and vaccination. Almost all cases of vaccination which progress unfavourably, may be traced to some previously unhealthy condition of the humors or secretions. So that a characteristic feature of the exanthemata is "absence of predisposing causes."

"All miasms of animal origin are capable of attaching themselves to fomites, and (provided they be excluded from the air) of retaining their communicating property for a considerable length of time. This great law of nature is the foundation of that important practical measure—quarantine. It is a law of universal application. Tinea capitis spreads by means of hats, combs, and brushes; Egyptian ophthalmia, by towels and sponges; small-pox and typhus, by clothes and bedding; plague, by personal apparel and old rags. Some would persuade us that merchandise, which, *ex necessitate rei*, could never have been near the chambers of the sick, or handled by others than by men in health, may also communicate contagion; but I believe this doctrine to be opposed to every principle in sound pathology."

From the well-known fact that medical men very seldom communicate the seeds of disease, Dr. Haygarth disbelieved in the doctrine of communication by fomites. That fact certainly proves how exceedingly volatile contagious miasms are, and how short an exposure to the air deprives them of noxious quality. The term of forty days originally judged necessary for the security of the community is founded on utter ignorance of the laws of morbid poisons. As the incubative stage of plague never exceeds seven days, so one week of quarantine is sufficient, and two weeks should satisfy the most scrupulous anxiety. It may be observed that all fomites or harbourers of contagion, are substances of a rough surface, or downy texture. Wool, cotton, leather, every kind of apparel, are the substances to be most guarded against. Metallic substances are considered incapable of harbouring contagion.

The sixth and last character of the exanthemata is drawn from their occurrence as *Epidemics*, a term which simply expresses the fact of the spreading of a disease among the people without reference to the precise

*mode* of communication. Some diseases accordingly are contagious but not epidemic, as ophthalmia, gonorrhœa and porrigo; whilst some are epidemic, but not contagious, as catarrh, diarrhœa and pneumonia. Again some diseases are both epidemic and contagious, as small-pox, measles, scarlet fever, typhus, plague, and probably cholera. With respect to the nature of the origin of epidemic diseases various opinions have been entertained. The present most approved theory of epidemic influence attributes everything to the atmosphere, but neither the thermometer, nor the barometer, nor the hygrometer, nor the electro-meter, aid us in our researches. Statistical research holds out the fairest prospect of our attaining to truth in this recondite branch of pathology. Ten diseases are placed by Mr. Farr in the category of epidemic maladies—namely, small-pox, measles, scarlatina, and hooping cough, the four great epidemics, together with croup, thrush, diarrhœa, dysentery, cholera, and influenza.

Though little is known with respect to the ultimate cause of epidemic visitation, still there are certain laws with reference to the diffusion of epidemics, which are sufficiently well established. Thus it rarely happens that two diseases are epidemic at the same time in the same district. Some exceptions, however, to this law have been observed. In 1839, both small-pox and measles were epidemic in England and Wales. It has been generally observed that epidemics are unusually severe when they first appear in any country, or are renewed after a long interval of time. Our author next considers the principles which are to guide us in the general management of the eruptive fevers. First, he lays it down that an exanthema cannot be cut short. It has been six, eight, or twelve days breeding, and so must run its course. The legitimate objects of treatment are to lessen inordinate constitutional tumult, to subdue plethora, and to check accidental congestions and complications. The great objects of treatment, in fact, in these disorders, are less directed to the specific malady than to those congestions and superadded affections by which the steady march of the exanthem is impeded. Hundreds of cases in these diseases may be conducted to a close without a grain of medicine; because, in such cases, the febrile action, or zymotic process goes on quietly, being neither too violent on the one hand, nor, on the other, deficient in the necessary power. To give active medicine here is hurtful. It deranges nature. But the case is different when the febrile commotion or effervescence is inordinately violent, as when small-pox is ushered in with phrenitis, measles with epistaxis, scarlatina, with excessive angina. Purgatives, leeches, cold lotions, bleeding from the arm, may then be required. On the other hand, should the vis vitæ fail, should the first effect of the poison be to reduce the powers of life so low that the disorder cannot develop its regular series of phases, when the extremities are cold, the eruption tardy, when syncope occurs, or actual collapse is threatened, then is the time for stimulants. Great caution is necessary in treating those cases, where debility is left behind after an attack of exanthematous disease. The difficulty consists in distinguishing real from apparent debility.

In his THIRD LECTURE, the author discusses *The Early History and Phenomena of Small Pox.*

The Greeks and Romans knew nothing of small-pox. The first notice of a disease that looks at all like it, occurs in a chapter of Procopius, "De Bello Persico" (lib. 11. ch. 22), where he describes a dreadful pestilence which began at Pelusium, in Egypt, about the year 544, and spread in two directions, towards Alexandria on the one side, and Palestine on the other—a very short time afterwards very unequivocal traces of small-pox are to be met with in the countries bordering on the Red Sea. Mr. Bruce, the celebrated Abyssinian Traveller, wishes to fix the first epidemic of small-pox to the era 522, which corresponds sufficiently near to the date of this plague described by Procopius. Rhayes (900) is the first writer who mentions small-pox; he gave a clear and full description of it. From the East small-pox travelled to the West, and appears to have reached England towards the close of the ninth century. This pestilence reached America about 1527. The ravages of this disease, dreadful as they are in temperate climates, are still more so in tropical. Coming to the year 1640, when the mode of treating fevers by the hot or sweating system had attained its acme, our author gives us a specimen of this mode of practice, extracted from the writings of Diemerbroek, a Dutch physician and professor. This practice was more especially applied to small-pox.

"Keep the patient," says Diemerbroek, "in a chamber close shut. If it be Winter, let the air be corrected by large fires. Take care that no cold gets to the patient's bed. Cover him over with blankets. Red blankets have always been preferred—not that the colour is material—but because, in the times of our ancestors, all the best, thickest, and warmest blankets, were dyed red. Never shift the patient's linen till after the fourteenth day, for fear of striking in the pock, to the irrecoverable ruin of the patient. Far better is it to let the patient bear with the stench, than to let him change his linen, and thus be the cause of his own death. Nevertheless, if a change be absolutely necessary, be sure that he puts on the foul linen that he put off before he fell sick, and, above all things, take care that this supply of semi-clean linen be well warmed. Sudorific expulsives are, in the mean time, to be given plentifully, such as treacle, pearls and saffron."

This is a sketch of the method of expelling the peccant humours in fever by perspiration, and such was the state in which Sydenham found the practice of medicine in 1667. No one can justly appreciate the vast merits of Sydenham, unless he is thoroughly acquainted with the writings of physicians during the first half of the seventeenth century. The next great epoch in the history of small-pox is that of inoculation. This was practised for the first time at Constantinople about the year 1700. To Lady Mary Wortley Montague the world is indebted for the introduction of this splendid improvement into medical practice. In 1746 the Small Pox Hospital was founded to enable the poor to participate in a benefit hitherto confined to the rich. In 1798 Dr. Jenner announced the discovery of vaccination. We now pass on to notice some of the more prominent phenomena of small-pox, commencing with the incubative stage or period. This period admits of some latitude. The extremes may be stated at ten and sixteen days. With respect to the eruptive fever of small-pox, it is characterised by the usual evidences of pyrexia—quick pulse, hot skin, pains of back and limbs, scanty and high-coloured urine, and restlessness. In deciding whether such a fever be variolous we are further assisted by other symptoms; and among these there may be mentioned—1, Intense sickness at stomach, accompanied by tenderness of the epigastrium on pressure—the vomiting yields when the eruption appears. 2. By pain of the back and loins. 3. By the occurrence of severe encephalic symptoms; severe headache, stupor or delirium; somnolency or epileptic fits are noticed to occur in children. 4. Syncope and great prostration of strength. 5. Great anxiety of the præcordia, deep sighing and dyspnœa. Our author now directs attention to the character of the variolous eruption, and in a special manner to the external character and internal structure of the variolous pimple and pustule—to the colour of the areola, &c.

He also directs attention to the implication of certain of the mucous membranes in the progress of small-pox. In a great proportion of confluent, and in some semi-confluent cases, the mucous membrane of all those parts to which the atmospheric air gets access, (the nose, mouth, and trachea) is occupied with eruption—sometimes distinct, more generally confluent. The early symptoms occasioned by this mucous complication are as follows: Numerous white points appear on the tongue, palate, and velum pendulum. Hoarseness and alteration of the voice indicate that the same condition extends to the mucous membrane of the larynx and trachea—there is great pain in swallowing, and, in bad cases, cough and dyspnœa.

The ulterior effects of this mucous complication become very important. The œdematous thickening of the larynx and the swollen condition of the tracheal membrane have by the eighth day materially impeded the free access of air to the lungs, and the consequences appear in every part of the circulating system. The areola is not crimson. The vesicles on the extremities never acquire any inflammatory areola. On the trunk the areola is dark or claret-coloured. The vesicles do not acuminate, but lie flat. At length, the brain becomes affected. A low muttering delirium is observed, as the waves of ill-oxygenated blood begin to circulate. The tongue swells and assumes a purple hue. The bladder loses its contractile power. The extremities become cold, and the patient dies.

The implication of the cellular membrane is next described with its various phenomena and consequences, and then that of the nervous system. He then considers the petechial form of small-pox,—and next as the disease becomes complicated with gangrene, ophthalmia, and affections of internal organs, together with the appearances on dissection.

We shall now make a few remarks on the diagnosis of small-pox. The diseases with which, after the occurrence of the eruptive fever, small-pox may be confounded, are measles, febrile lichen, varicella, and secondary syphilis.

1. The papulæ of small-pox are firmer than those of measles. They feel granular under the finger. In measles, too, there is accompanying cough and watering of the eyes. Further, in small-pox, forty-eight hours from rigor to eruption; in measles seventy-two.

2. Febrile lichen is the disease from which small-pox, at its onset, is with most difficulty distinguished. The aspect of the eruption is in both cases nearly alike. The surest and safest grounds of diagnosis are based on the interval which has elapsed from rigor to eruption, and the mode in which the eruption has developed itself. In febrile lichen twenty-four hours elapse from sickening to eruption ; in small-pox, forty-eight. Small-pox almost always appears first on the face. The eruption of lichen is developed, from the first, uniformly over the head and trunk. Besides all this, we must enquire into the prior history of the patient, and the character and course of the incubation.

3. The diagnosis between small-pox and a form of secondary syphilis, in which the eruption passes through the several grades of papulæ, vesicle and pustule, is to be effected by careful enquiry into the whole history of the case and close observation of the progress of the disease.

To follow our author farther through the subjects of these lectures, interesting though they confessedly are, would be altogether foreign to the object of our Journal, For full information on the interesting details contained in them we must refer our readers to the work itself, assuring them that an attentive perusal of its valuable contents will amply compensate them for their time and trouble. The book, in fact, is precisely the one we should have expected from the pen of an author so well versed in the pathology of exanthematous disease as Dr. G. Gregory is well known to be.

